# Understanding Concerns about COVID-19 and Vaccination: Perspectives from Kidney Transplant Recipients

**DOI:** 10.3390/vaccines11071134

**Published:** 2023-06-22

**Authors:** Sarah R. MacEwan, Alice A. Gaughan, Graham N. Dixon, Ramona G. Olvera, Willi L. Tarver, Saurabh Rahurkar, Laura J. Rush, Austin D. Schenk, Jack Stevens, Ann Scheck McAlearney

**Affiliations:** 1 Division of General Internal Medicine, College of Medicine, The Ohio State University, Columbus, OH 43210, USA; sarah.macewan@osumc.edu; 2 The Center for the Advancement of Team Science, Analytics, and Systems Thinking (CATALYST), College of Medicine, The Ohio State University, Columbus, OH 43210, USA; alice.gaughan@osumc.edu (A.A.G.); ramona.olvera@osumc.edu (R.G.O.); willi.tarver@osumc.edu (W.L.T.); saurabh.rahurkar@osumc.edu (S.R.); laura.rush@osumc.edu (L.J.R.); 3 School of Communications, College of Arts and Sciences, The Ohio State University, Columbus, OH 43210, USA; dixon.716@osu.edu; 4 Division of Cancer Prevention and Control, Department of Internal Medicine, The Ohio State University Comprehensive Cancer Center, The Ohio State University, Columbus, OH 43210, USA; 5 Department of Biomedical Informatics, College of Medicine, The Ohio State University, Columbus, OH 43210, USA; 6 Division of Transplantation, Department of Surgery, The Ohio State University, Columbus, OH 43210, USA; austin.schenk@osumc.edu; 7 Department of Pediatrics, The Abigail Wexner Research Institute at Nationwide Children’s Hospital, Columbus, OH 43205, USA; jack.stevens@nationwidechildrens.org; 8 Department of Family and Community Medicine, College of Medicine, The Ohio State University, Columbus, OH 43210, USA

**Keywords:** COVID-19, vaccination, vaccine hesitancy, trust, kidney transplantation

## Abstract

The COVID-19 pandemic poses a significant risk for immunosuppressed groups such as transplant patients. The purpose of this study was to improve our understanding of the impact of the COVID-19 pandemic on kidney transplant recipients, including their views on COVID-19 vaccination. Semi-structured interviews were conducted from December 2021 to August 2022 with 38 kidney transplant recipients who had an appointment with their transplant care team within the previous 6 months. We used qualitative thematic analysis to characterize the perspectives of interviewees. Regardless of COVID-19 vaccination status, most interviewees reported utilizing public health measures such as masking, hand washing, and avoiding crowds to protect themselves against COVID-19. Vaccinated interviewees (*n* = 31) noted that they chose to receive a COVID-19 vaccine because of their increased risk due to their immunocompromised state. For unvaccinated interviewees (*n* = 7), reasons for not receiving a COVID-19 vaccine included concerns about the safety and efficacy of the vaccine. Both vaccinated and unvaccinated interviewees expressed concerns about the lack of adequate testing of the vaccine in transplant patients and questioned if the vaccine might have unknown side effects for transplant recipients. Regardless of the vaccination status, most interviewees noted having trust in their healthcare team. Interviewees also described interpersonal tensions that arose during the pandemic, many of which surrounded vaccination and other preventive measures that were important to participants to protect their health. Together, these data demonstrate differing concerns and experiences related to the COVID-19 pandemic for vaccinated and unvaccinated transplant recipients. These findings highlight the unique needs of transplant recipients and reveal opportunities to support this vulnerable patient population in efforts to protect their health as the COVID-19 pandemic evolves.

## 1. Introduction

Kidney transplant recipients (KTRs) face physical, social, and psychological challenges to managing their health due to their treatment burden [1], subsequent immunocompromised state [2], and life-long medication management [3]. The COVID-19 pandemic presented additional challenges to KTRs as their immunosuppression put them at an increased risk of experiencing severe illness due to a COVID-19 infection. Solid organ transplant recipients in general [4] and KTRs specifically [5] have been shown to experience a higher risk of mortality from COVID-19 infection. While KTRs experienced a reduced relative mortality risk with the rise of the Omicron variant, similar to the general public, their immunocompromised state and high incidence of comorbidities still contributed to their continued high risk of COVID-19 infection and subsequent severe illness [6]. The COVID-19 pandemic also put KTRs at risk by disrupting access to ongoing healthcare that is important for transplant self-management [7]. KTRs have noted experiencing increased stress during the COVID-19 pandemic related to factors such as living in isolation [8], delayed medical appointments [9], fear of transplant failure [8], and loss of income or insurance [9].

For many individuals, COVID-19 vaccination presents an opportunity to protect oneself against infection and illness. Nevertheless, early clinical trials of COVID-19 vaccines did not include transplant patients [10]. Early research indicated general safety of mRNA COVID vaccination in solid organ transplant recipients [11], but KTRs who received the initial two doses of an mRNA vaccine produced a weaker immune response than immunocompetent individuals [12]. In a comparison among a small number of solid organ transplant recipients, vaccination with a viral vector vaccine induced an even weaker immune response in comparison to an mRNA vaccine [13]. A third dose of an mRNA vaccine was shown to increase immune response [14]. This led to the recommendation of an additional dose of the vaccine for this population [15], as well as vaccination guidelines provided for transplant recipients from the US Centers for Disease Control and Prevention [16]. Research is ongoing to identify strategies to best protect KTRs through vaccination or other prophylactic alternatives [17]. However, like the general population, not all KTRs have chosen to receive the COVID-19 vaccine where vaccine acceptance and refusal have been assessed in urban [18] and rural KTR populations [7]. While more is known about attitudes toward COVID-19 vaccination in the general population [19] both prior to [20] and following vaccine availability [21], less is known about these attitudes among the kidney transplant population specifically. Furthermore, there is little knowledge of the differences in pandemic experiences between vaccinated and unvaccinated KTRs. This exploratory study aimed to fill these gaps in knowledge through one-on-one interviews with individuals in this vulnerable population. Understanding these perspectives can provide valuable insight to inform clinical strategies that help protect and support these patients while COVID-19 remains a threat to public health. 

## 2. Materials and Methods

### 2.1. Study Setting and Population

We conducted interviews with KTRs receiving healthcare at The Ohio State University Wexner Medical Center (OSUWMC) in Columbus, Ohio. Patients who were at least 18 years old, had received a kidney transplant at any time in the past, had attended an appointment with their transplant team at OSUWMC within the last 6 months, and were fluent in English were eligible to participate in the study. We restricted the study population to English speakers for two reasons: (1) over 95% of kidney transplant patients at OSUWMC are English-speaking, and (2) our research team members trained in qualitative interviewing are English-speaking. As the vast majority of our study population were English speakers, we do not believe we introduced bias in understanding the perceptions of this population. 

The interviews took place between December 2021 and August 2022, and aimed to understand how KTRs were impacted by the COVID-19 pandemic. During the time of the study, COVID-19 vaccinations made by Pfizer-BioNTech (Mainz, Germany), Moderna (Cambridge, MA, USA), and Johnson & Johnson Janssen (Leiden, Netherlands), were readily available. This study was approved by The Ohio State University’s Institutional Review Board.

### 2.2. Study Design, Data Collection, and Interview Procedures

Eligible KTRs received a recruitment email from the study team and were provided with information about the study. Interested patients were then contacted by the study team and scheduled for interviews. As most kidney transplant patients at OSUWMC provided an email address that is listed in their electronic medical record, we do not believe we introduced significant bias by limiting our recruitment to a study population having an email address. We did offer interviews both by phone and videoconference, to limit bias related to the use of technology available to complete the interview. All interviewees provided verbal informed consent prior to participating in the study. One-on-one interviews were conducted by phone or videoconference using a semi-structured interview guide (see Supplementary File S1) that asked questions about the impact of the COVID-19 pandemic, including perspectives about COVID-19 vaccines. The interviews were audio-recorded, transcribed verbatim, and de-identified for analysis. 

### 2.3. Data Analysis

We used deductive dominant thematic analysis [22,23] to code and analyze interview transcripts. This allowed for the categorization of data based on general themes derived from the interview guide, as well as identification of emergent themes using the constant comparative approach. Preliminary coding was performed by a single team member. Two study team members then ensured consistent application of the codes and identified and applied emergent codes across all transcripts. ATLAS.ti software (ATLAS.ti Scientific Software Development; Berlin, Germany) was used to support our qualitative coding and the analysis process. The quotations presented have been lightly edited for clarity to remove filler words (e.g., uh, um, yeah, like). 

We conducted interviews until we reached saturation in our thematic analysis. Saturation in qualitative data analysis means that collecting more data will not lead to more information, as the themes being characterized are repeated in subsequent interviews and no new themes emerge. In the context of this study, this involved hearing similar responses across participants. Given this as a standard for rigorous qualitative research, we are confident that we interviewed a sufficient number of participants to reach data saturation and achieve the validity of our results.

## 3. Results

### 3.1. Interviewee Characteristics

Thirty-eight KTRs, with a mean age of 57 years (range: 31–76) completed interviews (Table 1). The interviews lasted an average of 32 min (range: 13–53 min). The interviewees were on average 7 years post-transplantation (range: 1 month–25 years). The ratio of men to women was even (50%). Eighty-two percent (*n* = 31) of participants reported having received at least one dose of the COVID-19 vaccine; 32% (*n* = 12) of participants self-reported one or more instances of COVID-19 infection. Of the participants who reported COVID-19 infection, 75% reported that they had been vaccinated.

### 3.2. Perspectives about COVID-19 Precautions

Participants shared their general perspectives about the precautions they took to protect themselves during the COVID-19 pandemic (Table 2). Notably, both vaccinated and unvaccinated participants shared similar feelings about preventing exposure to COVID-19 using preventive measures. Both groups discussed using prevention methods such as masks, handwashing, sanitizer, and social distancing. For example, one vaccinated participant shared: “I still wear a mask everywhere. I still try to distance myself from people, especially when I go to the grocery store. I’m selective to where I go. I won’t go into any crowded areas.“ Similarly, an unvaccinated interviewee explained: “I usually don’t touch doors. I don’t touch people’s hands. I don’t hug very often. I always use sanitizer, wash my hands, wear a mask in the doctor’s office. But that’s pretty much what I do”. For those participants who had received their kidney transplant before the pandemic, some described their preventive behaviors in response to the COVID-19 pandemic as a continuation of precautions they were already taking due to their vulnerability from their transplant. However, some unvaccinated participants mentioned becoming more relaxed with public health measures over time: “I stayed inside as much as possible to begin with, but I still ended up getting it, but I got a very mild case. We use masks and hand washing. I’m a lot more relaxed about it now than I was last year”. 

### 3.3. Perspectives about COVID-19 Vaccination

Participants also shared a variety of perspectives about COVID-19 vaccination (Table 3). Those who chose to receive a COVID-19 vaccine predominantly explained that their choice was motivated by their increased risk related to their immunocompromised state. One vaccinated participant described: “Just having the kidney transplant. Just common sense to do it just because of my low immune system. I thought it would be a better idea to get it than not get it”. On the other hand, some unvaccinated participants believed that the potential side effects of the vaccine outweighed the risk of COVID-19 infection. One unvaccinated participant shared: “Why get really sick when you never get the actual disease? ...You’re playing Russian roulette either way. And I would rather not take the chance of getting the vaccine and even getting semi-sick from just the vaccine while my body builds immunity to it. So, I just didn’t do it”. Despite the differences in their decisions to receive a COVID-19 vaccine, both vaccinated and unvaccinated participants described concerns about vaccination. These concerns included unknown side-effects of vaccination and lack of evidence about vaccine efficacy, specifically for transplant recipients. One vaccinated participant explained their concern: “I’m an immunocompromised patient. I didn’t want to get it and have stuff happen. So, I ended up getting it after long deliberations. Yeah, but you have to, I still have my doubts and stuff about it”. For some participants, these concerns were enough to convince them not to receive a vaccine, as described by one unvaccinated participant: “Mostly my objection to it all was that they had never done what I consider adequate and proper testing on just the transplant population to examine the potential downside side effects, long-term effects, actual protection for someone suppressed like myself and with a donor kidney”. Two unvaccinated participants shared that they would receive a COVID-19 vaccine if it had been required for kidney transplantation. As one of these participants explained, “Let’s just say that my kidney would fail, and I could possibly need another kidney and that would come up. If I was made to do it, I would do it”. The other similarly noted, “I think [hospital] was one of the places that you didn’t have to require it for a transplant. I know there was some other hospitals that you had to have it to get it. Now if in that case, if say [provider name], says, ‘Okay, you have to get a COVID vaccine,’ of course I would get it, for just the transplant. I would just bite the bullet and get it”. 

### 3.4. Perspectives about Healthcare Team

Participants also commented about their healthcare teams, including describing recommendations regarding vaccination they had received from care teams and the trust they placed in their healthcare providers (Table 4). Both vaccinated and unvaccinated participants overwhelmingly reported that their healthcare providers recommended that they receive a COVID-19 vaccine. One unvaccinated participant described the recommendation they received: “Just that people should get the vaccine just because it will further protect me. And I mean, that really just sums it up. Because I was so immunocompromised, they really suggested it”. Nevertheless, among unvaccinated participants, these recommendations were sometimes met with skepticism. As one participant explained, their provider told them, “People who have transplants are at more high risk. And that they would recommend it, that I get one because they believe it’s helpful for people with a weakened immune system to have the vaccine in case you do have to fight COVID-19 or anything like that. They always want me to get the flu shot. Well, I did it for a couple years, and every time I got super sick”. Some unvaccinated participants noted that their care team recommended the vaccine but did not press it. As one participant described, “They suggested to me to get the vaccine. But when I say no, they don’t get knock-down, drag out with me. And that’s a good thing because it wouldn’t do any good”.

Both vaccinated and unvaccinated participants predominantly reported trust in their care teams. One vaccinated participant shared, “Well, I’ve always trusted in the medical people that serve me. They tell me do this and I do it. I can’t tell you ever in my life, where a medical person has ever told me to do something that was detrimental to my health. They’ve always been on my side”. Similarly, one unvaccinated participant commented that their care team was a trusted source of information: “I’ve never had to doubt them. And other sources you have to doubt sometimes. That’s what made [healthcare provider] trustworthy”. This included seeing care teams as a trusted source of information about COVID-19 vaccination. As one unvaccinated participated described: “I trust my primary care doctor an awful lot about things because he’s reasoned with me. He’s the only one that’s ever made me think about getting a vaccine”.

While many participants in both groups described trusting their healthcare teams, some still expressed doubts. These included doubts about evidence in the medical community regarding vaccination within the transplant population. One vaccinated participant questioned: “I still don’t think that the medical profession fully understands, for example, how protected am I? Is it 60 percent, 70 percent, 40 percent? I don’t think anybody can answer that question”. Other doubts were fueled by misinformation, which impacted their trust in medical providers in general. As one unvaccinated participant described: “When I asked a doctor, well how many of your patients have gotten the vaccine? And the answer, lots. That’s absolutely no help at all. When you’re searching and you’re hearing the vaccine injuries and the political football that it is and you hear that hospitals and doctors get x amount of dollars if they pushed the vaccine. And you know when the CDC is paid for by Pfizer. I mean, it’s been extremely hard to trust doctors through this period”.

### 3.5. Perspectives about Interpersonal Tensions around COVID-19 Prevention

Finally, participants described interpersonal tensions that arose during the pandemic, many of which surrounded vaccination and other preventive measures that were important to participants to protect their health (Table 5). For both vaccinated and unvaccinated participants, these tensions included self-implemented changes, with respect to engaging with family members and others. Vaccinated and unvaccinated participants described frustration when others did not recognize the increased health risks for them as transplant patients, and failed to adjust behaviors accordingly, for example, by avoiding hugging or hand shaking. For vaccinated participants, self-imposed changes in behavior also included avoiding unvaccinated individuals. As one vaccinated participant described: “It’s weighing the options and [trying] to figure out who’s going to be there in the family. I pretty much know who has been vaccinated and who hasn’t. I just have to react accordingly. If there is someone there that is unvaccinated, you just don’t go”. 

Tensions also surfaced regarding vaccination decisions among family members and friends. While some participants described avoiding discussions about vaccination, others described how these discussions led to conflict. Some unvaccinated participants described tension caused by their feeling condemned for their personal decision not to be vaccinated. As one participant described, “Our whole family’s split in two, just like most families in this country. …‘You’re an idiot. You’re going to kill Mom or Grandma.’ Well, the non-vaxers have to just shut up. The non-vaxers are just quietly making their choice, while the vaxers are condemning everybody else. It just adds into the politics of why we refused it in the first place”. For vaccinated participants, tensions were reportedly higher when they perceived others’ decisions to not get vaccinated as putting them at a greater risk. For example, one participant explained, “I’ve told friends and relatives that they’re stupid, selfish, and irresponsible. …They’re the ones that will suffer. But that could affect me. And that pisses me off”. One vaccinated participant suggested it might be helpful to have resources that could facilitate discussions about COVID-19 vaccination for transplant patients: “I think sometimes, too, from someone with a compromised immune system, it might be helpful to have some talking points and explaining how I’m at a higher risk and why those in my circle getting vaccinated can help protect people like myself that are immunocompromised, which sounds very selfish, and I realize that. But I think just having some talking points to help me explain that to family members or friends could be a really helpful thing to have”. 

## 4. Discussion

KTRs have been a high-risk patient population throughout the COVID-19 pandemic due to their immunocompromised state. This status has led to questions about transplant patients’ abilities to fight COVID-19 infection, as well as about their abilities to mount immune responses from vaccination [24]. While growing research is increasing our knowledge about COVID-19 infection and the COVID-19 vaccine responses in immunocompromised individuals [25,26], less attention has been paid to the experiences of transplant patients during the pandemic as they navigate the risks of this public health crisis [16]. Our study served to fill this knowledge gap by exploring the perspectives of KTRs, particularly related to KTRs’ perspectives about COVID-19 vaccination and the differences in pandemic experiences between those who were vaccinated or unvaccinated.

Despite the differences in vaccination status, many KTRs in our study shared similar perspectives about preventive behaviors and concerns related to their health risks during the COVID-19 pandemic. Although unvaccinated participants noted reasons for not receiving a COVID-19 vaccine that are aligned with the views expressed by the general population [27], they also reported concerns specific to transplant recipients, such as lack of evidence for vaccine efficacy in this population and concerns about side-effects due to their immunocompromised status. Similar concerns have been reported from a quantitative analysis of surveys of KTRs in the United States prior to [28] and after the release of COVID-19 vaccines [7], and among quantitative and qualitative analyses of KTR perspectives in other countries including Australia [29], Croatia [30], Italy [31], and Singapore [32]. Several studies utilizing survey analyses highlighted concerns about COVID-19 vaccination impacting transplant rejection [28,32], but this was mentioned infrequently by our study participants.

The differences among the perspectives of vaccinated and unvaccinated participants in our study may stem from factors that have been shown to be associated with vaccination acceptance and refusal in other populations, such as perceptions of COVID-19 risk [33]. Our study population included individuals who varied with respect to the time elapsed since receiving their kidney transplant, and this may have impacted their perceptions of the risk about both COVID-19 vaccination and the impact of the COVID-19 pandemic. Medical mistrust has also been demonstrated to be a factor impacting the differences in vaccine hesitancy [34], but both vaccinated and unvaccinated KTRs in our study reported high trust in their healthcare team, which may differ from the decline in medical trust that has been seen in the general population [35]. In practice, this trusted relationship may open opportunities for discussion about vaccination with transplant patients.

Some unvaccinated KTRs likely represent so-called “fence sitters”, whose more moderate vaccine-hesitant views lack the extreme hardline positions found in the anti-vaccine community. Coupled with higher trust in healthcare providers and greater personal experience in healthcare settings, unvaccinated transplant recipients may be more amenable to nudges that encourage COVID-19 vaccination. For example, interventions that highlight the consequences of non-vaccination have been effective at improving attitudes toward vaccination for individuals with a moderate degree of vaccine-hesitant views [36]. On the other hand, when vaccine hesitancy represents one’s social identity and is shaped by conspiratorial thinking—a characteristic common in the anti-vaccine community—interventions typically encounter stiff resistance and are ultimately unsuccessful [37]. Given their high medical trust and unique healthcare experiences, unvaccinated KTRs may therefore benefit from vaccine messaging via trusted medical sources that highlight the consequences of remaining unvaccinated. Nurses may be particularly effective in encouraging vaccination among KTRs, as the general public rates nurses as having the greatest honesty and ethical standards among 18 different professions [38]. Coupling this messaging with targeted campaigns to increase vaccination among KTRs and those considering kidney transplant as a treatment option [18,39] may further support vaccination among this vulnerable population. 

Finally, participants noted experiencing interpersonal tensions around preventive behaviors during the COVID-19 pandemic, including vaccination, as well as behaviors such as social distancing. Both vaccinated and unvaccinated participants described frustration when the behaviors of others did not align with their preferences and expectations. These findings present an opportunity to support KTRs by providing language that can be used to communicate how preventive health behaviors are important to protect the health of immunocompromised populations. Several organizations, including the World Health Organization, U.S. Department of Health and Human Services, and UNICEF, have compiled tips for discussing COVID-19 vaccination with friends and family [40,41,42]. Tailoring talking points to acknowledge the risks and concerns specific to KTRs could better facilitate these difficult discussions for transplant patients.

Our work has several limitations. First, our study was conducted at a single medical center. Second, less than 20% of our interviewees were unvaccinated, so perspectives of the unvaccinated were less prevalent among our interviewees. Third, we did not collect clinical or sociodemographic information about our participants, which prevented analyses based on COVID-19 vaccine type, severity of COVID-19 illness, or patient characteristics. In light of our limitations, these findings demonstrate opportunities for future research, including the inclusion of study populations across healthcare systems, and performing analyses that link patient perspectives to clinical and sociodemographic characteristics. Lastly, our study was conducted prior to the availability of the COVID-19 bivalent booster. As the recommendations for different types of vaccines and boosters continue to evolve as the COVID-19 pandemic transitions to an endemic phase, continued research is also warranted to understand the current perceptions and attitudes of transplant recipients that may differ from those observed at the time of this study.

## 5. Conclusions

This work advances our understanding of the impact of the COVID-19 pandemic on KTRs. Although KTRs were generally vigilant about protecting themselves from COVID-19 by using public health measures, and most accepted guidance from their healthcare team, some KTRs remained skeptical about COVID-19 vaccination. Reasons for not getting vaccinated mirrored concerns often voiced by immunocompetent people, while additional concerns were expressed about the lack of research on immunosuppressed populations. Navigating social interactions with individuals who were not willing to comply with public health measures was a noted source of stress for many interviewees. Our findings highlight the necessity for more research throughout the translational spectrum to enable healthcare providers to address the unique needs and concerns of immunocompromised patients.

## Figures and Tables

**Table 1:** Participant characteristics.

Participant Characteristics	Mean (±SD) or *n* (%)
Age (years)	
Mean (±SD)	57 (±12)
Range	31–76
Gender	
Male	19 (50)
Female	19 (50)
COVID-19 Vaccine Status	
Vaccinated	31 (82)
Unvaccinated	7 (18)
COVID-19 Infection	
One or more instances	12 (32)
None	26 (68)

**Table 2:** Perspectives about COVID-19 precautions.

Vaccination Status	Comments from KTRs
Vaccinated	Especially given the medications I’m on, with immunosuppressants and all that, I pretty much mask up in public and sanitize my hands a lot.
	It’s just more awareness, you know, making sure I use a sanitizer before I play with my grandchildren. We have hand sanitizer in the car. So, every time we get back in the car, we make sure we use it. We’re still having our groceries picked up instead of going through the grocery store. I think a heightened awareness of our situation is appropriate.
	I try to stay home. I recently started working again outside of the home and I wear my mask all the time. I don’t have the people over to my house. My husband has people over to the house and one of my requirements is they have to wear masks here.
Unvaccinated	Distance, you know, keeping enough distance is key.
	Having been a transplant patient and having gone through a couple years of dialysis, I already had some pretty strict personal hygiene, and that included everything that went away with COVID. I hadn’t even been to a buffet for years.
	It was to pretty much stay isolated or away from anywhere we thought was a threat, especially myself. So, I did not venture out very much, surely not in crowds, not in a supermarket, church, things like that. So that was mine. Mine was complete withdrawal.

**Table 3:** Perspectives about COVID-19 vaccination.

Vaccination Status	Comments from KTRs
Vaccinated	I didn’t want to get sick and risk losing my transplant or anything worse than that.
	My feeling was that I have a lot to risk if I get COVID. I’m already immunosuppressed. …So, I decided that I would take the chance and so that maybe I wouldn’t have to take a chance for COVID. I would take a chance to get the COVID vaccine.
	I did have concerns about side effects, that it was not, it was my understanding, it wasn’t fully tested on transplant patients. But to me, I felt the alternative of getting COVID was worse.
Unvaccinated	With all the health issues I have, it terrifies me. …I looked at ingredient lists and side effects and just a whole different slew of stuff. And I’m just too afraid of what it could do to me.
	My mom, she’s immunocompromised as well, she has multiple sclerosis. So, she was really worried about getting it [vaccine] and because of how it would react with her body. And I was the same way. …They’re talking about how people die and all this. Instantly people just instantly die on the news, which I don’t know if it’s true or not. But just the fact that they’re saying that, it’s just like, “Okay, is this really a hundred percent like they say so?” And those people would die, and they don’t have anything wrong them, but they still die. So, I’m like, man, I’m immunocompromised. It’s just like a higher risk.
	I got personal and asked a lot of doctors and a lot nurses, have you had the vaccine? Yes. Have you had COVID since? Yes. So, I have not seen or have not been convinced that it stops you from getting it.

**Table 4:** Perspectives about healthcare teams.

Vaccination Status	Comments from KTRs
Vaccinated	The transplant team and my primary care provider, I would hope that they wouldn’t steer me wrong or give me false information. …I have faith in them because I’m still living. I had a transplant. I had to have faith in them for that. I’m still around.
	They [care team] didn’t know if it [a COVID vaccine] would work for someone taking immunosuppressants.
	At the very beginning, it was like, “Well, we’re [the care team] not sure. They haven’t done a lot of testing with transplant patients, so we’re not sure how well you’ll respond to it, but you know, it can’t hurt”.
Unvaccinated	I tell [my doctor] to convince me [to get the vaccine], and he came the closest to talking to me in an intelligent way that explained things to me in a way that wasn’t just, do it because I say to do it, don’t just do it because I do it. And he explained it to me a little scientifically, and he made some sense to me.
	I trust my folks at [hospital] quite fully. My doctors and stuff at [hospital], yes. But I do follow up in my own research.
	Just you should get the vaccine. And you know, how many got it? A lot. That was about the extent of it. My family doctor, the local one, he was really emotional about it. “Oh, I’m seeing these people dying in the hospital”. And two weeks later after he’s vaccinated and he’s boosted, he’s sick with it.

**Table 5:** Perspectives about interpersonal tensions.

Vaccination Status	Comments from KTRs
Vaccinated	So that goes back to the vaccination; the same people who are not getting vaccinated are a lot more loose with their COVID protocols. So, you know, I’ve made the decision during these cold winter months, like I’m sorry, I just can’t see them. I can’t take that risk to see them indoors.
	Everybody now has taken off their masks and have kind of resumed life as normal. …And that concerns me because the vaccinations don’t make you invulnerable to the disease or to the sickness. …I can’t just walk around like nothing’s happened, that the vaccination didn’t suddenly, you know, totally protect me from getting COVID. Now, I think some people feel that they’ve been vaccinated, that’s how they feel. And that’s fine. But they don’t have compromised immune systems is what I’m getting at.
	It was kind of challenging because this Thanksgiving, we generally host. And so feelings were hurt because I asked my sister and brother-in-law, who aren’t vaccinated, if they would go get tested and they were offended that I just asked them.
Unvaccinated	I don’t think [people] realize the seriousness sometimes and the vulnerability of the transplant recipient and they’re a little careless. But my immediate family get it, and they’re very careful, cautious, and they open doors for me.
	So, it’s a strict divide. There’s the vaxers and the anti-vaxers or the non-vaxers. I’m not anti-vax. I’m non-vax. And to me, the vaxers are the ruder, crueler, meaner ones. That’s my perception.
	People want to hug me and shake my hand and I just don’t. And in that kind of conversation, I’m the stuck up one. Don’t touch me. We’ll get really frustrated if we know somebody’s been exposed to something and they’re insensitive about that, concerning me.

## Data Availability

Due to participant privacy concerns, the data presented in this study are not publicly available.

## Supplementary Materials

The following supporting information can be downloaded at: https://www.mdpi.com/article/10.3390/vaccines11071134/s1, File S1: INTERVIEW GUIDE.
