# Supplementary material for: Understanding Concerns about COVID-19 and Vaccination: Perspectives from Kidney Transplant Recipients
Source: Vaccines (Basel). 2023 Jun 22;11(7):1134. doi: 10.3390/vaccines11071134 (PMC10386736; doi:10.3390/vaccines11071134)
Supplement: Supplementary file 1 [file vaccines-11-01134-s001.zip › vaccines-2414537-supplementary.pdf]

File S1: INTERVIEW GUIDE

Section S1: Introduction and background

- To start, for our records, what is your age?

- In which county do you live?

Section S2: Perspectives on COVID-19 as a Transplant Recipient

- With the emergence of COVID-19, did you have a household plan or an approach to protect you and your family (or household unit) from infection?

    - What was in the plan (or what did you do)?
        - Probe: How are you using masks, social distancing, handwashing, quarantine, or isolation as a part of your plan?
        - Did you have any trouble getting the supplies to protect yourselves?

    - How has your approach changed over time?

    - Have others' approaches or attitudes changed over time?

Section S3: Perspectives on COVID-19 viral testing

- Have you ever had a viral test for COVID-19 (e.g., the nasal swab – this tells you if you are actively infected with the coronavirus)?

If Yes:

- Where did you get tested?
    - Was this required by your employer or your decision?
        - *If respondent's decision:* Why did you decide to get tested? (e.g., close contact tested positive)

- What did you think about the test and the testing process?

- Did you experience any barriers to getting tested? (e.g., information, need to have symptoms, confusion about type of test)
    - What were they?
    - Did anyone in your household get tested? How was their experience in comparison to yours?

- How were your test results communicated to you? (e.g., through a portal—have to log in or not?)
    - How soon did you receive your results?
    - What information was included with your results?
    - Was there anything about the test results that you did not understand?
    - Was there any other information you wish you had received after getting tested? (e.g., quarantining guidelines, more clarity about what to do next)

If No:

- Have you ever heard of a viral test for COVID-19?
    - Where did you get or hear information about testing?

- Has your doctor offered you the possibility to get tested?

- Do you know where you can get tested?

- Would you know what to do with your test results?

- Would the test results change your behavior? (e.g., social distancing, mask wearing)

- What has prevented you from being tested?

- Do you have other concerns about getting tested for COVID-19?

Section S4: Perspectives on COVID-19 serological testing

- Have you ever heard of a serological test for COVID-19 (e.g., a blood test that tells you if you had an antibody response to the coronavirus, for example, you were previously infected and your immune system cleared the virus)?

    - What do you know about this testing option?
    - Where did you learn about it?

- Have you ever had a serological test for COVID-19?

If Yes:

- Why did you get the test?

- Where were you tested?

- How were the test results communicated to you? (e.g., through a portal—have to log in or not?)
    - What information was included with your results?

- Do you understand what to do with your results?

- Did the test results change your behavior? (e.g., social distancing, mask wearing)

- Did you experience any barriers or challenges to getting tested? (e.g., cost)

- Is there anything that would have made getting tested easier? (e.g., cost)

- Did you receive any follow-up after you got tested?
    - Can you please explain what that process looked like?
      OR
    - If you needed follow-up regarding your test results, where would you go?

- Is there anything you wish was done differently during follow-up?

If No:

- Why have you not been tested?
- If you wanted or needed to be tested, do you know where you would go?
- If you were tested, would you understand what to do with those results?
- Is there anything you would like to know more about regarding serological testing?
- Would having a plan from your doctor regarding how and when to get tested be helpful?
  - If yes, how would it be helpful?
  - If no, why would it not be helpful?
- What resources would be helpful to getting tested?

Section S5: Confusion around COVID-19 guidance

- Where have you received information about COVID-19 exposure and transmission? (e.g., your employer, your doctor)
  - How was that information provided to you?
  - What type of guidance have you received?
- Are you confused at all about guidance around mask wearing, contact tracing, etc.?
- Have you had to contract trace any of your close contacts?
  - What was your experience with that?
- Are there other areas where you wish there had been better guidance around COVID-19? (e.g., how to quarantine, the need to contact trace)
- Have you received information about the downstream health consequences of COVID-19?
  - How was that information provided to you?

Section S6: Perspectives on a COVID-19 vaccine

- Now that a vaccine has become available for COVID-19, are you planning to get one?
  - If yes, why?
  - If no, why not?
- Have you talked to any coworkers about getting a vaccine?
- Do you have any concerns about getting a vaccine for COVID-19?
- What types of messages do you think should be promoted about the vaccine, etc.?

Section S7: Messaging and communications about COVID-19

- What types of messages do you think should be promoted about COVID-19, the need for testing, vaccinations, etc.?

- To get the message out about COVID-19 in your community, what are the best ways to communicate information?
  - Social media (e.g., FaceBook, NextDoor)
  - Word-of-mouth?
  - Advertisements, billboards, newspaper, radio?
  - Health care provider recommendation?
  - Community health workers, patient navigators?
  - Print materials?
  - Faith community?
  - Other places? (e.g., barbershops, hair dressers, etc.)

- What concerns do you have about health and safety since the emergence of COVID-19?

- Do you have any concerns about misinformation (i.e., incorrect information) regarding COVID-19 in your community? About the vaccines?

- What have you done when information about COVID-19 has rapidly changed?
  - Has this been a concern? Confusing?

Section S8: Perspectives on impact of COVID-19

- How has the COVID-19 pandemic affected your access to or experiences with healthcare?

- Have you delayed any medical appointments, treatments, or procedures (related to your cancer or transplant treatment or other healthcare needs) due to the COVID-19 pandemic?

- Have you have any concerns about your medical care during the COVID-19 pandemic?

- What are the greatest challenges that you see now with this pandemic? (e.g., fatigue, vaccine, supplies, economic impact)

- What are you concerned about looking farther ahead?

- Can you describe the impact of this pandemic on you?

- How do you think the pandemic has impacted the mental health of you or your family members/household contacts?

INTERVIEW CLOSURE AND FOLLOW-UP

- Is there anything else we should know or that you would like to tell us regarding viral testing, serological testing, or vaccination for COVID-19?

Thank you so much for your time and participation. Your comments were extremely helpful.
